# Supplementary material for: The effect of Dnaaf5 gene dosage on primary ciliary dyskinesia phenotypes
Source: JCI Insight. 2023 Jun 8;8(11):e168836. doi: 10.1172/jci.insight.168836 (PMC10393236; doi:10.1172/jci.insight.168836)
Supplement: Supplemental table 1 [file jciinsight-8-168836-s125.pdf]

**Supplement Table 1:** Known pathogenic variants in *DNAAF5*

| Variant | Location* | Inheritance | Reference | ClinVar ID |
|---|---|---|---|---|
| c.2394C>T (p.Arg792Ter) | HEAT 10 | Ter, homozygous | Unpublished | |
| c.2384T>C (p.Leu795Pro) | HEAT 10 | Missense, Homozygous | PMID:23040496 | 39684 |
| c.2374C>T (p.Arg792Ter) | HEAT 10 | Ter, unknown inheritance | Unpublished | 1454678 |
| c.2432-1G>C | HEAT 10 | splice mutant, Homozygous | PMID:**25232951** | |
| c.2353_2352AG, (p.Ser785fs) | HEAT 10 | FS, Homozygous | PMID:**29358401** | 241202 |
| c.2346C>G (p.Tyr782Ter) | HEAT 10 | Ter, unknown inheritance | Not reported | 454860 |
| c.2108_2114delinsCCACCCTGGGT (p.Met703fs) | HEAT 8 | FS, compound heterozygous | Unpublished‡ | 454858 |
| c.2083-1G>C | | homozygous | Unpublished | |
| c.2013_2028delinsATGGCC (p.Ala672fs) | HEAT 8 | FS, unknown inheritance | Unpublished | 649289 |
| c.1784-1G>T | | splice acceptor, unknown inheritance | Unpublished | 454854 |
| c.1777_1778del (p.Gln593fs) | HEAT 5-6 | FS, unknown inheritance | Unpublished | 1453546 |
| c.1731G>T (p.Trp577Cys) | HEAT 6-7 | Missense, compound heterozygous | Unpublished‡ | |
| c.1499G>T (p.C500F) | HEAT 5-6 | 1.Missense, homozygous 2.compound heterozygous | PMID:29358401 PMID:29363216† | |
| c.1142_1147delAGCTGC (p.Gln381_Leu382del) | HEAT6 | In-frame deletion, homozygous | Unpublished | |
| c.948G>A (p.Trp316Ter) | HEAT 4 | nonsense, unknown inheritance | Unpublished | 663589 |
| c.926G>A (p.Trp309Ter) | HEAT 4 | nonsense, unknown inheritance | Unpublished | 651485 |
| c.943C>T (p.Gln315Ter) | HEAT 4 | Nonsense, unknown inheritance | Unpublished | 1432731 |
| c.745del (p.His249fs) | HEAT 3 | FS, unknown inheritance | Unpublished | 1433639 |
| c.489_498del (p.His163fs) | HEAT 1-2 | FS, unknown inheritance | Unpublished | 454865 |
| c.363_373del (p.Ala122fs) | HEAT 1-2 | FS, unknown inheritance | Unpublished | 454864 |
| c.8C>T (p.Ala3Val) | N-terminus | Missense, homozygous | Unpublished | |
| c.208C>T (p.Gln70Ter) | C-terminus | nonsense, unknown inheritance | Unpublished | 843828 |
| c.55dup (p.Ala19fs) | N-terminus | FS, compound heterozygous | PMID:29363216† | 689528 |

*Location, HEAT-repeat motif site of the variant
†Reported as compound heterozygous (p.Ala1fs/Cys500Phe)
‡Reported as compound heterozygous (Met703FS/ Trp577Cys)
Abbreviations, FS, frame shift; Ter, termination
